# Expression of ribosomopathy genes during *Xenopus tropicalis* embryogenesis

**DOI:** 10.1186/s12861-016-0138-5

**Published:** 2016-10-26

**Authors:** Andrew Robson, Nick D. L. Owens, Susan J. Baserga, Mustafa K. Khokha, John N. Griffin

**Affiliations:** 1 Program in Vertebrate Developmental Biology, Departments of Pediatrics and Genetics, Yale University School of Medicine, 333 Cedar Street, New Haven, CT 06510 USA; 2 The Francis Crick Institute, Mill Hill Laboratory, The Ridgeway, London, NW7 1AA UK; 3 Departments of Genetics, Molecular Biophysics and Biochemistry, and Therapeutic Radiology, Yale University School of Medicine, New Haven, CT 06520 USA

**Keywords:** Ribosome, Development, Diamond-Blackfan anemia, North American Childhood Cirrhosis, TCOF1, RPL, RPS, UTP, Ribosome biogenesis, Ribosomopathy, *Xenopus*

## Abstract

**Background:**

Because ribosomes are ubiquitously required for protein production, it was long assumed that any inherited defect in ribosome manufacture would be embryonically lethal. However, several human congenital diseases have been found to be associated with mutations in ribosome biogenesis factors. Surprisingly, despite the global requirement for ribosomes, these “ribosomopathies” are characterized by distinct and tissue specific phenotypes. The reasons for such tissue proclivity in ribosomopathies remain mysterious but may include differential expression of ribosome biogenesis factors in distinct tissues.

**Methods:**

Here we use *in situ* hybridization of labeled antisense mRNA probes and ultra high temporal resolution RNA-Seq data to examine and compare expression of 13 disease associated ribosome biogenesis factors at six key stages in *Xenopus tropicalis* development.

**Results:**

Rather than being ubiquitously expressed during development, mRNAs of all examined ribosome biogenesis factors were highly enriched in specific tissues, including the cranial neural crest and ventral blood islands. Interestingly, expression of ribosome biogenesis factors demonstrates clear differences in timing, transcript number and tissue localization.

**Conclusion:**

Ribosome biogenesis factor expression is more spatiotemporally regulated during embryonic development than previously expected and correlates closely with many of the common ribosomopathy phenotypes. Our findings provide information on the dynamic use of ribosome production machinery components during development and advance our understanding of their roles in disease.

**Electronic supplementary material:**

The online version of this article (doi:10.1186/s12861-016-0138-5) contains supplementary material, which is available to authorized users.

## Background

Ribosomes translate genetically encoded information into proteins. Their production is a ubiquitous, carefully regulated, and energetically expensive cellular process. It is so essential to life that it alone accounts for over 60 % of total transcription in eukaryotic cells [1, 2]. While it was long assumed that any defect in this fundamental process would be incompatible with life, mutations affecting ribosome production have now been identified in several human congenital disorders (ribosomopathies) and in animal models [3–15]. Despite all arising from abnormalities in the ubiquitous process of ribosome biogenesis, these diseases exhibit unexpectedly unique phenotypes (Table 1) [1, 16–18]. The reason for this tissue proclivity remains mysterious but its existence hints at a fundamental cell type specificity in ribosome biology during development.

**Table 1:** Diseases of Ribosome Biogenesis

Ribosomopathy	Proteins	Function	Phenotype
Diamond-Blackfan Anemia	RSP7, RPS10, RPS17, RPS19, RPS24, RPS26, RPS27, RPS29	Processing of 18S rRNA	anemia, bone marrow failure, craniofacial and limb defects, cancer predisposition
	RPL5, RPL11, RPL15, RPL26, RPL27, RPL31, RPL35A	Processing LSSu rRNA	anemia, bone marrow failure, craniofacial and limb defects, cancer predisposition
5q-Syndrome	RPS14	18S rRNA processing	anemia, bone marrow failure, myelodysplastic syndrome, cancer predisposition
Treacher-Collins Syndrome	TCOF1	rDNA transcription & 18S processing	Craniofacial
North American Indian Childhood Cirrhosis	UTP4 (formerly Cirhin)	18S rRNA maturation	Billary cirrhosis
Bowen-Conradi Syndrome	EMG1	Maturation of SSU	Growth retardation, psychomotor delay, skeletal
Isolated Congenital Asplenia	RPSA	Maturation of SSU	Spleen loss
X-linked subtype of dyskeratosis congenita	Dyskerin	Linked to H/ACA snoRNA and TERC	Variable, reticulated hyperpigmentation of skin, nail dystrophy, leukoplakia, bone marrow defects
Shwachman-Diamond Syndrome	SBDS	Maturation and export of LSU	Growth retardation, exocrine pancrease insufficiency, skeletal and hematologic defects, cancer predisposition
Alopecia, neurological and endocrinopathy syndrome	RBM28	Maturation of LSU	Growth retardation, impaired motor skills, mental retardation, hair loss, skeletal and skin abnormalities, adrenal defect.

**Table 2:** Sites of enriched ribosome biogenesis factor expression

		Ribosome biogenesis factors												
		*rbm28*	*sbds*	*tcof1*	*utp4*	*rps7*	*rps14*	*rps17*	*rps19*	*rps29*	*rpl15*	*rpl26*	*rpl35a*	*rpl38*
Sites of enriched expressions	4 Cell	+	+	+	+	-	+	+	+	+	-	-	-	+
	Gastrula	+	+	+	+	+	+	+	+	+	-	-	+	+
	Anterior neural folds	+	+	+	+	+	+	+	+	+	-	+	+	+
	Neural fold border	+	+	+	+	+	+	+	+	+	-	+	+	+
	Migrating neural crest	+	+	+	+	+	+	+	+	+	-	+	+	+
	Brain	+	+	+	+	+	+	+	+	+	+	+	+	+
	Specific mid/hind brain border	+	-	+	+	-	-	-	-	-	-	-	-	-
	Hyoid arch	+	+	+	+	+	+	+	+	+	+	+	+	+
	Mandibular arch	+	+	+	+	+	+	+	+	+	+	+	+	+
	Neural tube	+	+	+	+	+	+	+	+	+	+	+	+	+
	Paraxial muscle	+	-	+	-	-	-	+	+	+	+	+	-	+
	Eye	+	+	+	+	+	+	+	+	+	+	+	+	+
	Ear	+	+	+	+	+	+	+	+	+	+	+	+	+
	Heart/liver region	+	+	+	+	+	+	+	+	+	-	+	+	+
	Pronepheros	-	-	-	-	-	+	+	-	-	+	+	+	+
	Ventral mesoderm/blood islands	+	-	+	+	-	-	+	+	+	+	+	+	+

**Fig. 1:**
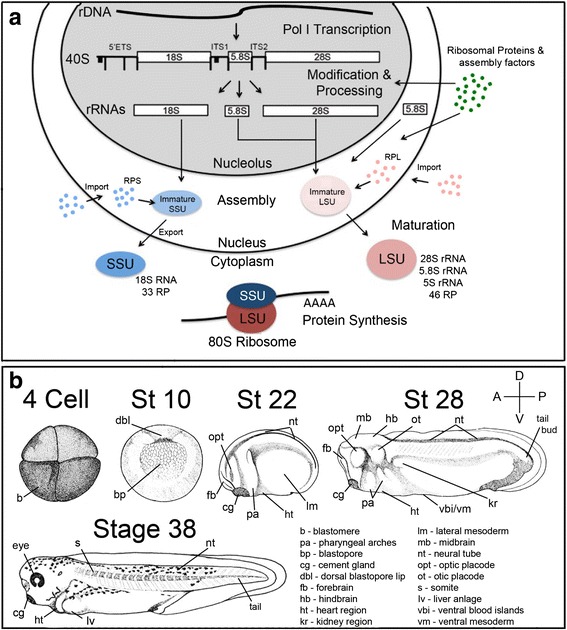
**a** Schematic of ribosome biogenesis. The rDNA is transcribed by RNA polymerase I and the resulting rRNA is processed in the nucleolus before being incorporated into the large ribosomal subunit (LSU) or small ribosomal subunit (SSU). **b** Schematic of *X. tropicalis* embryos at examined stages. Major anatomical features are labeled. A: anterior, P: posterior, D: dorsal, R: ventral

Ribosome production is a complex process. Briefly summarized, each 80S ribosome is composed of two ribonucleoprotein subcomplexes. The large 60s subunit (LSU) is composed of the 28S, 5.8S and 5S ribosomal RNAs (rRNAs) and 46 associated ribosomal proteins (RPL), while the small 40S subunit (SSU) contains the 18S rRNA and 33 ribosomal proteins (RPS, Fig. 1a) [1, 18]. Production of these subunits begins in the nucleolus with the transcription of the polycistronic pre-ribosomal RNA (rRNA) by RNA polymerase I (RNAPI). This large precursor rRNA then undergoes numerous sequential cleavages, modifications and foldings to ultimately produce the mature 18S rRNA, 5.8S and 28S rRNAs. The 5S rRNA is transcribed separately by RNA polymerase III (Fig. 1a). Correct transcription and processing of the rRNAs and their assembly into the ribosomal subcomplexes requires the concerted action of all three RNA polymerases, 75 small nucleolar RNAs (snoRNAs), and over 200 ribosome biogenesis factors [1, 16, 19–21]. Each of these biogenesis factors must also be transcribed by RNA polymerase II, translated by cytoplasmic ribosomes and localized correctly to carry out its function in the nucleolus, nucleus or cytoplasm (Fig. 1a) [1, 16, 18].

A number of human diseases are now associated with defects in ribosome production (Table 1). For example, lesions in at least 15 different ribosomal proteins (RP) underlie **Diamond-Blackfan anemia** (DBA, OMIM# 105650, RPL and RPS mutations) [22–31]. These anomalies affect pre-rRNA processing and assembly of the SSU or LSU, and typically result in bone marrow failure. Craniofacial, growth and limb defects are also commonly present in patients [16, 22, 25]. Mutations in the ribosome biogenesis factor hUTP4 (formerly called Cirhin), cause **North American Indian Childhood Cirrhosis** (NAIC, OMIM# 604901, UTP4 mutations), a form of biliary cirrhosis [32, 33]. Without a liver transplant in childhood, this condition is fatal. In **Shwachman-Bodian-Diamond syndrome** (SBDS, OMIM# 260400), patients present with pancreatic failure, bone marrow defects and skeletal abnormalities caused by mutations in the SBDS protein that impair maturation of the large ribosomal subunit [34–36]. **Treacher-Collins syndrome** (TCS, OMIM# 154500, typically TCOF1 mutations) is characterized primarily by craniofacial defects [1, 3, 5, 16, 18, 37–39]. Other major ribosomopathies include **Chromosome 5q- syndrome** (5q, OMIM# 153550, acquired RPS14 mutations, macrocytic anemia) [1, 16, 40], **Alopecia, Neurological and Endocrinopathy syndrome** (ANE, OMIM# 612079, RBM28 mutations) [41], and **isolated congenital asplenia** (ICA, OMIM# 271400, RPSA mutations), that specifically impairs spleen development [42]. Numerous additional confirmed and suspected ribosomopathies exist (Table 1). While some of the ribosomopathies have defects in common (e.g. anemia, growth retardation, craniofacial defects, increased risk of cancer), these diseases are clinically distinct and difficult to treat. Understanding their etiology is a first step towards improving treatment options and patient prognosis.

Why defects in the ubiquitous production of ribosomes lead to tissue specific phenotypes remains a fascinating and clinically important question. One possibility is that the affected tissues are simply rapidly dividing and are thus more sensitive to reduced ribosome numbers. However, this does not account for the non-overlapping phenotypes observed in these syndromes, or explain why some highly active populations appear unaffected. Other proposed mechanisms include 1) selective translation of mRNAs with internal ribosome entry sites and 2) extra ribosomal functions specific to each protein [16, 43–48]. One intriguing possibility that may explain the diverse effects of ribosomal protein abnormality is that ribosomes are tailored to translate specific mRNAs, or have particular properties in different cell types. Changes in ribosomal composition and biogenesis may then affect these activities. In support, reduction of Rpl38 in mice can alter the composition of *Hox* gene mRNAs translation and embryonic patterning, without reducing overall protein synthesis [6]. Furthermore, ribosomal gene mRNA expression exhibits significant variation across tissues [6, 46, 47, 49–51]. Such variation may result in differential ribosome production and activity. This idea of specialized ribosomes challenges our notion of ribosomes as monolithic entities and may suggest a fundamental mechanism of translational regulation. However, a comprehensive spatiotemporal understanding of ribosomopathy associated gene expression during development is not known, which is essential to correlate with the tissue proclivity observed in ribosomopathies.

As a preliminary step in examining this issue, we analyzed the expression of thirteen distinct ribosome biogenesis factors (*rps7, 14, 17, 19, 29, rpl15, 26, 35a, 38, tcof1, rbm28, sbds* and *utp4*) using whole mount *in situ* hybridization and ultra high temporal resolution RNA-Seq data. Mutations in twelve of these factors are implicated in six phenotypically distinct human conditions. We found that expression of these genes is greatly upregulated in specific tissues during development, particularly in the cranial neural crest (CNC) and ventral mesoderm, and broadly correlates with common ribosome disease phenotypes. While the general pattern of expression is similar for all examined genes, unexpected differences do exist. Overall our study reveals extensive spatiotemporal regulation of ribosome biogenesis factor expression during embryonic development.

## Methods

### *Xenopus* embryos

*X. tropicalis* were maintained and cared for in our aquatics facility, in accordance with Yale University Institutional Animal Care and Use Committee protocols. Embryos were produced by in vitro fertilization and raised to appropriate stages in 1/9MR + gentamycin [52].

### Whole-mount *in situ* hybridization

Full length clones for; *rbm28*, TTpA003i23; *rpl15*, IMAGE: 7606910; *rpl26*, TNeu089l11; *rpl35a*, TNeu023b09; *rpl38*, TNeu074p13; *rps7*, TNeu091o12; *rps14*, IMAGE: 8896584; *rps17*, TNeu101k22; *rps19*, TNeu108f02; *rps29,* TGas050g16; sbds, TGas062n22; *tcof1* TEgg112k11, and *upt4,* TGas051c03 were obtained in pCS107 vector from the Sanger/Wellcome Trust *Xenopus tropicalis* cDNA libraries. Digoxigenin-labeled antisense mRNA probes were in vitro transcribed with T7 High Yield RNA Synthesis Kit (E2040S) from New England Biolabs. Embryos were collected at desired stages, fixed in MEMFA for 1–2 h at room temperature and dehydrated into 100 % ETOH. Whole mount *in situ* hybridization was performed as described previously [4, 53]. Embryos were stained with BM Purple and examined after equilibration in 100 % glycerol.

### High temporal resolution RNA-Seq

Gene expression trajectories were compared using our previously published RNA-seq data [54] (GEO: GSE65785), which reports gene expression in absolute transcripts per embryo. *X. tropicalis* v7.1 models as used in [54] for *rpl15, rpl26, rpl35a, rpl38, rps7, rps17, rps19* were found to be incorrect, and were corrected with reference to reads and gene models from *X. tropicalis* v4 assembly. Corrected gene models are available in Additional file 1. Gene expression abundances in absolute transcripts per embryo were re-quantified with improved models as described in [54].

## Results

To study expression of ribosome biogenesis factors during embryonic development, we examined six key developmental stages: 3, 10, 15, 20–22, 28–30, and 36–40 (Fig. 1b). These stages mark critical time points during embryogenesis, including maternal RNA inheritance, germ layer specification, morphogenesis and organogenesis. We examined gene expression in numerous anatomical structures including the neural tube, streams of migrating neural crest, pharyngeal arches, heart, eye, ventral mesoderm, and pronephros (described in Fig. 1b). *In situ* hybridizations using digoxigenin labeled sense mRNA probes for each gene were carried out as controls (Additional file 2).

### Expression of ribosome biogenesis factors

We began by examining the expression patterns of four ribosome biogenesis factors that have previously been implicated in phenotypically distinct human malformations, *tcof1, rbm28, sbds,* and *utp4*.

**Fig. 2:**
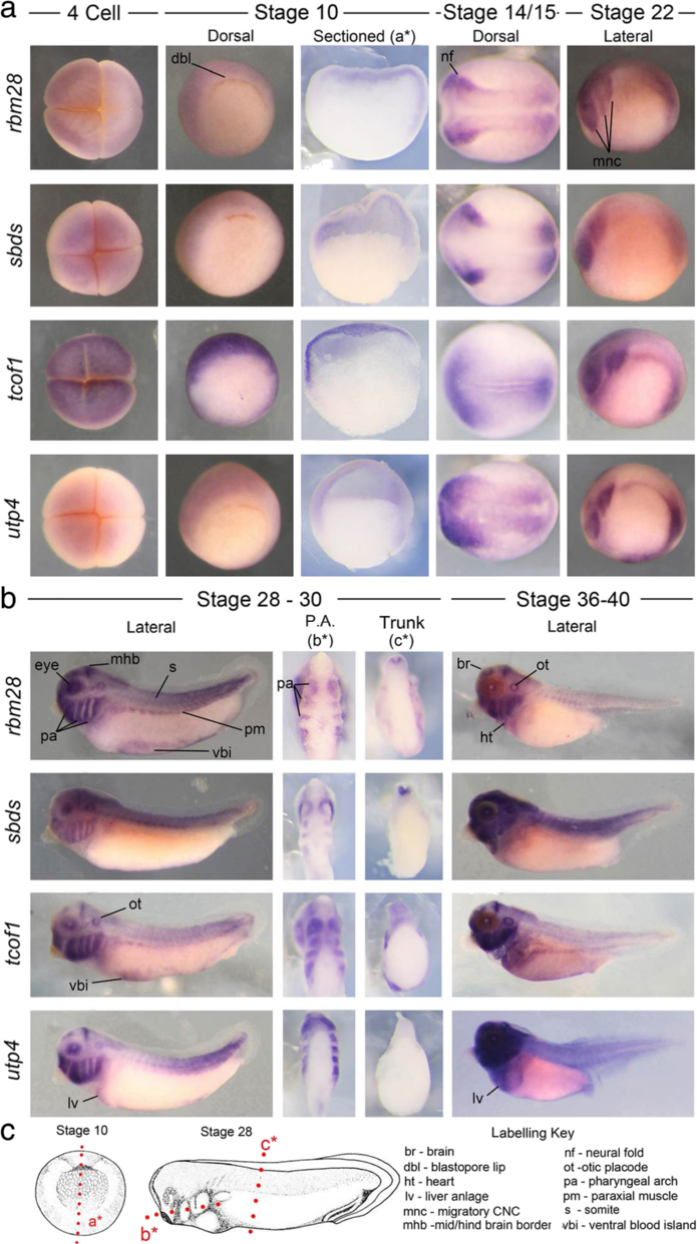
Expression of disease-linked ribosome biogenesis factors in *X. tropicalis* embryos. **a** Expression of all four genes was detected in the 4-cell and gastrulating embryo. Signal was strongly detected in the neural folds at stage 16 and in the migrating neural crest at stage 22. **b** At later stages expression of all factors was detected in the facial primordia and developing brain. Note the restricted expression at the mid–hindbrain border and forebrain. Expression was also typically detected in ventral mesoderm and hypaxial muscle precursor cells. *utp4* mRNA was strongly detected at the site of the future liver. **c** Red lines labeled a*, b* and c* in diagrams of stage 10 and 28 embryos represent the planes of section shown in **a** and **b**

With the exception of *tcof1*, signal for each factor was weakly detected at the 4-cell stage and in the animal pole surrounding the blastopore at stage 10. Interestingly, *tcof1* expression appeared much more robust in these tissues (Fig. 2a). At stage 16 expression of all genes was strongly associated with the anterior neural plate and neural fold borders where CNC form. At stage 22 transcripts were robustly detected in the migrating CNC and the posterior region of the neural tube. Expression was typically much weaker or absent in the intermediate regions of the neural tube. Uniquely, *utp4* transcripts were detected in the ventral mesoderm at this stage (Fig. 2a).

At stage 28 all four ribosome biogenesis factors were similarly expressed in the CNC populated craniofacial regions as well as the developing eye and otic placode (Fig. 2b). Interestingly, expression within the developing brain appeared strong and spatially restricted at the mid – hindbrain border (a crucial signaling center) and in discreet regions of the forebrain. Expression of *rbm28, tcof1* and *utp4* also appeared enriched in the posterior neural tube and tail bud, while expression of *sbds* was more uniform along the length of the neural tube. *tcof1 a*nd *rbm28* were also detected in hypaxial muscle and the ventral mesoderm/blood island region (Fig. 2b). Coronal sections through the craniofacial region revealed strong and specific expression of each gene within the mandibular, hyoid and branchial CNC populated pharyngeal arches, while transverse sections through the trunk confirmed expression in the neural tube (Fig. 2b). At stage 36–40 this precise spatial regulation of expression was largely maintained. *tcof1* and *rbm28* were expressed strongly in the CNC populated facial regions, the developing ear, and strongly in the mid-hindbrain and fore-midbrain boundaries. Their expression appeared significantly less robust, or absent, in other regions of the brain and neural tube. Interestingly, *sbds* was detected much more broadly throughout the craniofacial region and neural tube at these stages while *utp4* expression was greatly enriched specifically in the head. While all assayed genes were detected diffusely in the region of heart and liver at stage 36, *utp4* expression was observed in this region at stage 28 and was notably broad and robust here at stage 36–40 (Fig. 2b).

### Expression of *rps g*enes: *rps7, 14, 17, 19, 29*

We next examined the expression of several key RPS genes. *RPS* *7, 17, 19* and *29* are all associated with Diamond-Blackfan anemia, which is principally characterized by defects in bone marrow, craniofacial and limb development. *RPS14* mutations underlie 5q- syndrome, an acquired ribosomopathy that is characterized by macrocytic anemia and cancer predisposition.

**Fig. 3:**
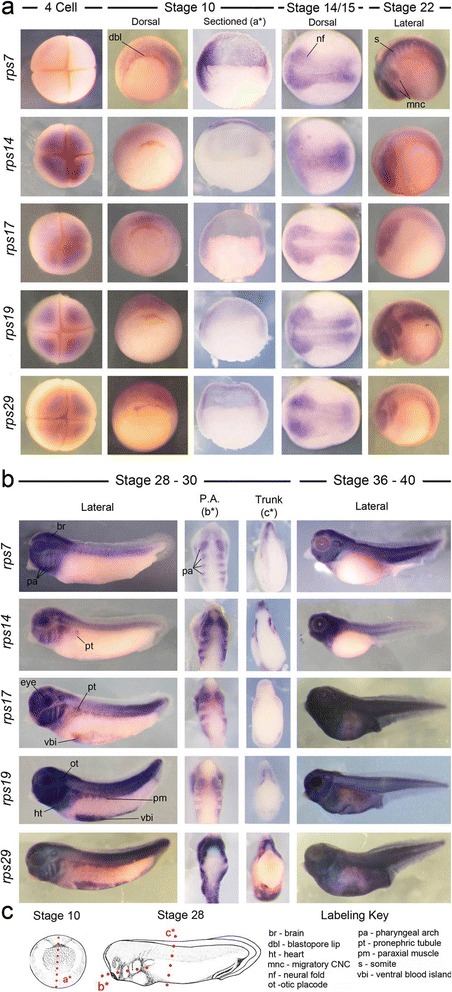
*rps* genes are dynamically expressed in development. **a** Expression of all five *rps* genes examined was observed in the stage 10 gastrula, stage 16 neural folds, and migrating CNC at stage 20–22. **b** Each gene was widely detected in the developing head at later stages (28–40), including all the pharyngeal arches, brain, eyes and ears. *rps17,* 19 and 29 were also robustly detected in the ventral mesoderm, while *rps7* was particularly defined in somites. With the exception of rps7, transcripts of all mRNAs were detected in the dorso-lateral plate mesodermal region where the pronephros and hypaxial muscle will form. **c** Red lines labeled a*, b* and c* in diagrams of stage 10 and 28 embryos represent the planes of section shown in **a** and **b**

At the 4-cell stage we detected variable amounts of maternal rps transcripts. *rps7* revealed little or no signal in the animal pole, while *rps14* signal was relatively strong. *rps17, 19* and *29* each displayed intermediate levels of staining (Fig. 3a). At stage 10, shortly after the maternal to zygotic transition, all RPS genes examined exhibited comparably diffuse signal around the blastopore of whole mount gastrulating embryos. Expression of rps7 appeared more robust in the animal tissues when embryos were sectioned (Fig. 3a). At stage 15 expression of all genes was detected within the neural folds and border regions where the CNC are being induced. At stages 18–22 the examined *rps* genes were expressed in the neural tube, developing brain and migrating neural crest. Interestingly, *rps7* transcripts were more highly detected in the somites when compared to other *rps* mRNAs (Fig. 3a).

At stage 28, all were robustly expressed throughout the craniofacial regions, including the CNC populated mandibular, hyoid, anterior and posterior pharyngeal arches, forebrain, midbrain, hindbrain, otic and optic placodes. Interestingly, we detected signal in the dorso-lateral plate mesodermal region where the pronephic tubule and hypaxial muscle will form in *rps14, 17, 19* and *29*, but this signal was not observed in *rps7* (Fig. 3b). However, *rps7* expression in the somites continued to be much more defined than that of other RPS genes. Robust expression of *rps17, 19* and *29* was also detected in the heart and ventral mesoderm/blood island regions at this stage suggesting a possible role in hematopoiesis, while expression of *rps7* was not evident in the ventral blood islands, and *rps14* was not detected in the heart or blood islands (Fig. 3b). By stages 36–40 *rps17, 19* and *29* were ubiquitously detected throughout the embryo, while *rps7* and *14* were absent from ventral regions.

### Expression of *rpl* genes*: rpl15, 26, 35a,* and *38*

Mutations in numerous *RPL* genes have also been implicated in DBA. Here we examine the developmental expression pattern of three such *rpl* genes (*15, 26 & 35a*). We also compare expression of *rpl38*, which has not yet been identified in human disease but heterozygous loss in mice results in anemia and skeletal malformations [6].

**Fig. 4:**
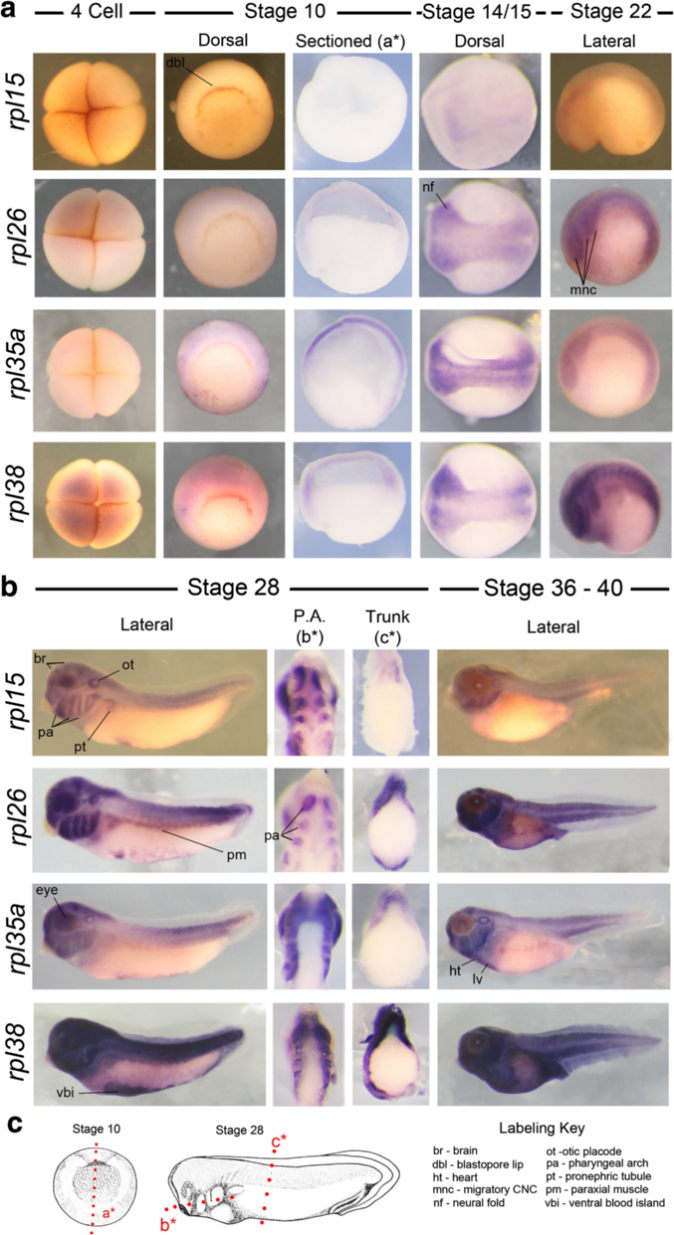
Expression of *rpl* genes. **a** *rpl* mRNA was weakly detected or absent at early stages of development but, with the exception of *rpl15*, became up-regulated in the neural folds and migratory neural crest from neurula stages onwards. **b** At later stages expression of all four *rpl* genes was evident broadly throughout the head, pharyngeal arches, neural tube and ventral mesoderm/blood islands. **c** Red lines labeled a*, b* and c* in diagrams of stage 10 and 28 embryos represent the planes of section shown in **a** and **b**

Expression of all assayed *rpl* genes was weak or absent at 4-cells. *rpl15* and *rpl26* expression was also undetected at gastrulation stages, while *rpl35a* and *rpl38* were clearly evident in the animal pole (Fig. 4a). Interestingly, while *rpl26, rpl35*a and *rpl38* transcripts were strongly detected in the neural folds at stage 16, *rpl15* remained absent. At stage 22 *rpl26, rpl35a* and *rpl38* mRNA were detected in the developing brain, neural tube and migrating neural crest (Fig. 4a). *rpl38* also exhibited very defined expression domains within the somites. Expression of *rpl15* continued to be very low or absent at this stage.

By stage 28, expression of all four *rpl* genes was strongly associated with the developing brain, neural tube, all pharyngeal arches, otic and optic placodes, developing heart field and ventral mesoderm/blood islands. This pattern was broadly maintained at stage 36–40. Expression of *rpl26* and *rpl38* appeared ubiquitous at these stages (Fig. 4b).

### High temporal resolution RNA-seq analysis

We previously created a genome-wide RNA-seq dataset with ultra high temporal resolution [54]. This dataset allows precise measurement of absolute transcripts per embryos, and comparison of expression dynamics for the entire transcriptome during *Xenopus* development. Here we utilized this rich resource to measure ribosome associated gene transcript kinetics in the embryo.

**Fig. 5:**
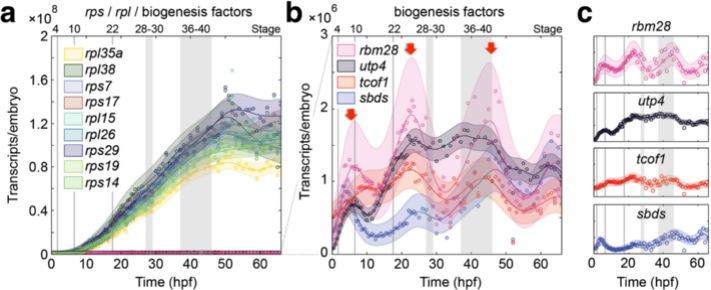
Dynamics of gene expression of poly(A) + mRNA in transcripts per embryo . **a** Plot of all examined genes. Note the relatively consistent dynamics but variably levels of of RP gene transcription. **b** Expression dynamics of ribosome biogenesis factors. While variable, three pronounced peaks in expression are evident during development (*red arrows*). **c** Expression of individual biogenesis factors

Ribosomopathy associated genes displayed significant variation in transcript numbers and expression dynamics throughout embryogenesis. While all RP genes examined exhibited a similar dynamic, evident as a continuous increase in expression as the embryo grows, there were pronounced differences in absolute transcript numbers (Fig. 5a). Ribosome biogenesis factor genes were expressed at lower levels than *rpl* or *rps* mRNAs and exhibited highly dynamic and regulated expression profiles (Fig. 5b). This was evident both in the number of transcripts and in the temporal regulation of expression (compare *sbds* and *rbm28* for example). Interestingly these biogenesis factors generally exhibited three, variably pronounced, peaks of expression during development suggesting increased biological demand at these stages (red arrows, Fig. 5b). The first peak occurs during early gastrulation stages as the embryo undergoes the complex cell movements and differentiations required to correctly establish the germ layers. The second peak occurs between stages 22–28, an extremely active developmental window marked by cell migrations, differentiation, rapid growth, and morphological change (Fig. 5b). Similarly, a third expression peak was evident at stage 36–45, a time of pronounced organogenesis and activity.

## Discussion

Ribosomes are generally thought of as monolithic cellular machines, ubiquitously expressed and functionally identically in every cell and tissue. However, an emerging body of research suggests that this is not the case. In particular, the diverse tissue specific phenotypes observed in human ribosomopathies and animal models point to an intriguing variation in requirements for ribosome biogenesis factors and/or ribosome biology during development [1, 4, 6, 7, 16, 25, 32, 36, 42, 46, 47, 50]. Here, we examined the developmental expression patterns of 13 ribosome-associated genes in *Xenopus*. With the exception of *rpl38*, the human orthologue of each of these has been implicated in disease. We found that ribosome-associated genes are not ubiquitously expressed during *Xenopus* development. Instead expression of components of the ribosome production machinery was highly spatiotemporally regulated, suggesting tissue specific activity.

All examined ribosome biogenesis factors were expressed in generally similar and dynamic spatial patterns. This included strong expression in the cranial neural crest, pharyngeal arches arches, eye and ears. Expression was also commonly detected within the developing brain, neural tube and ventral blood islands (summarized in Table 2). While overall patterns were similar, surprising differences were noted in both location and quantity of transcripts. For example, *rps7* transcripts were strongly detected in the somites at stage 22 but not in the developing kidney or hypaxial muscle at stage 28, while *rps14, 19* and *29* were the opposite. Even more striking was the clear restriction of ribosome biogenesi*s* factor expression compared to constituents of the LSU and SSU *(rps* and *rpl* genes). At stage 22 these biogenesis factors (Fig. 1) were typically greatly enriched in the anterior and posterior regions of the neural tube while *rps* and *rpl* transcripts were more evenly distributed. Moreover, at stage 28 their expression in the head was largely restricted to the CNC, eye, ear and mid-hindbrain boundary compared to the much broader craniofacial expression of *rpl* and *rps* genes (compare Fig. 2 vs. Figs. 3 and 4). Transcript number and expression kinetics also varied dramatically over time, hinting at unexpected stoichiometric relationships in ribosome production. Interestingly, the rhythmic expression observed in biogenesis factors, particularly *rbm28*, is similar to the dynamics of circadian genes. This observation fits well with previous reports that the circadian clock regulates ribosome biogenesis and mRNA translation [55]. These restricted and varied expression patterns are suggestive of tissue specific requirements and together with previous studies [6, 51, 55] suggest a greater dynamism of ribosome production genes than generally assumed.

The expression patterns observed also correlate closely with common ribosomopathy phenotypes. For example, craniofacial defects and macrocytic anemia are frequent features of ribosomopathies, particularly DBA and TCS [3, 5, 18, 22, 23, 37, 43]. We found expression of all 15 ribosome production genes to be strongly associated with the CNC and developing head. The sensitivity of the CNC to perturbations in ribosome production has been noted in both human patients and in animal models [3–5, 7], where it often appears to trigger a nucleolar stress response leading to increased apoptosis within the developing CNC and other tissues [4, 5, 7, 13, 45, 56–58]. The consistent and robust expression of all 15 examined ribosome associated genes in the CNC and head provides further evidence of a high ribosomal requirement in these tissues and suggest that ribosomal defects may contribute to unexplained craniofacial malformations. The majority of gene transcripts were also detected in the ventral mesoderm, in the region of the ventral blood islands. This compartment makes a critical contribution to the erythrocyte population in the developing tadpole [59, 60]. While bone marrow is not yet formed in the tadpole at the stages examined, this expression is consistent with a role in red blood cell development. Several other genes also overlapped with their clinical phenotypes. For example, *rbm28* expression in the developing brain and *utp4* expression at the site of the future liver correlate with disease phenotype.

However, later stage embryonic expression patterns alone do not accurately predict disease phenotype, as all examined genes display broadly similar expression but are associated with phenotypically distinct diseases. For example, we detected *utp4* in the craniofacial regions similar to *tcof1, rpl* and *rps* genes. However, its mutation in humans and knockdown in zebrafish lead specifically to a failure in liver development [1, 8, 32]. In comparison *tcof1* mutation is characterized by craniofacial malformations, while *rpl/rps* mutations often produce a broader range of abnormalities, including anemia, craniofacial and growth defects [1, 3, 5, 8, 10, 14–16, 22, 23, 32, 34, 37, 43]. Expression at earlier stages of development, or the availability of maternal derived protein, may partially explain this apparent paradox. Interestingly, our RNA-Seq and *in situ* data suggest relatively high levels of maternal *tcof1* transcript at early developmental times relative to *rps/rpl* genes. Thus, there may be sufficient *Tcof1* present to meet early developmental requirements, such as hematopoietic stem cell specification, but not later CNC demands. This early enrichment of *tcof1* and relative paucity of *rp* gene expression has also been observed in zebrafish [10, 25, 39], suggesting it is an evolutionary conserved feature. In future studies, it would be informative to further assay initial levels of maternal transcript or protein and determine if this correlates with the timing of phenotype.

The biological reasoning behind these restricted gene expression patterns, and their mechanistic contribution to disease remain unclear. It is possible that these tissues are highly active and require greater numbers of ribosomes, which in turn makes them more sensitive to ribosome biogenesis defects. However, it is then surprising that we do not see expression in other active and rapidly dividing tissues at these stages, or identical expression patterns for all the ribosome biogenesis factors examined. Several ribosome biogenesis factors and proteins have extra-ribosomal functions [16, 19, 44, 48, 61–63], or roles in selective translation of internal ribosome entry site mRNAs [16, 43, 46, 47, 64], which may also contribute to ribosomopathy phenotypes. “Specialized ribosomes”, tailored to preferentially translate specific mRNAs, or to have specific properties in particular environments are another possibility [6, 16, 17, 25, 46, 47, 50]. Support for this is found primarily in observations that ribosomal gene expression levels do vary between tissues [6, 7, 49, 51, 65, 66], and direct evidence of a regulatory role for individual RP proteins in selective mRNA translation and embryonic tissue patterning [6, 16, 43, 49, 50]. Furthermore, in *Dictyostelium* ribosome composition appears to shift with cell cycle [67, 68]. These findings suggest that ribosomes are not ubiquitously identical machines. Our observation of differential ribosome biogenesis factor expression during development provides additional support for this idea.

## Conclusions

While not new, the concept of specialized ribosomes is potentially a paradigm shifting advancement in our understanding of the fundamental interactions between ribosome biology, translation, cell cycle and embryonic development. Such topics are the focus of ongoing investigations and promise to greatly enhance our knowledge of a core biological process and a severe class of human diseases. In demonstrating the highly regulated and varied expression patterns of ribosomopathy genes during embryonic development, our results provide further support for the existence of tissue specific ribosomal requirements or functions, which may contribute to the startling tissue proclivity of ribosomopathies.

## Additional files

Additional file 1: Corrected *rps* and *rpl* gene models with coordinates against the *X. tropicalis* v7 genome assembly. (PDF 57 kb)
Additional file 2: Comparison of digoxigenin labeled anti-sense and sense mRNA probes for each ribosomopathy gene in stage 16 embryos. Dorsal and anterior views are shown. Note the strong expression detected in the neural plate by the anti-sense mRNA probes while the control sense probed embryos remains unstained. (JPG 922 kb)

## Abbreviations

CNC: Cranial neural crest
DBA: Diamond-Blackfan anemia
NAIC: North American Indian Childhood Cirrhosis
rbm28: RNA Binding Motif Protein 28
RP: Ribosomal protein
sbds: Shwachman-Bodian-Diamond Syndrome (Protein)
tcof1: Treacle ribosome biogenesis factor 1
