# Supplementary material for: Expression of ribosomopathy genes during Xenopus tropicalis embryogenesis
Source: BMC Dev Biol. 2016 Oct 26;16:38. doi: 10.1186/s12861-016-0138-5 (PMC5081970; doi:10.1186/s12861-016-0138-5)
Supplement: Additional file 1: — Corrected rps and rpl gene models with coordinates against the X. tropicalis v7 genome assembly. (PDF 57 kb) [file 12861_2016_138_MOESM1_ESM.pdf]

scaffold_7	NO	gene	54735692	54743620	.	+	.	ID=Xetro.N01056;Name=rps17;
scaffold_7	NO	mRNA	54735692	54743620	.	+	.	ID=Xetro.N01056.1;Name=rps17.3;Parent=Xetro.N01056;
scaffold_7	NO	exon	54735692	54735727	.	+	.	ID=Xetro.N01056.1.exon.1;Parent=Xetro.N01056.1;
scaffold_7	NO	five_prime_UTR	54735692	54735724	.	+	.	ID=Xetro.N01056.1.five_prime_UTR.1;Parent=Xetro.N01056.1;
scaffold_7	NO	CDS	54735725	54735727	.	+	0	ID=Xetro.N01056.1.CDS.1;Parent=Xetro.N01056.1;
scaffold_7	NO	exon	54737263	54737414	.	+	.	ID=Xetro.N01056.1.exon.2;Parent=Xetro.N01056.1;
scaffold_7	NO	CDS	54737263	54737414	.	+	0	ID=Xetro.N01056.1.CDS.2;Parent=Xetro.N01056.1;
scaffold_7	NO	exon	54737848	54737953	.	+	.	ID=Xetro.N01056.1.exon.3;Parent=Xetro.N01056.1;
scaffold_7	NO	CDS	54737848	54737953	.	+	1	ID=Xetro.N01056.1.CDS.3;Parent=Xetro.N01056.1;
scaffold_7	NO	exon	54739128	54739193	.	+	.	ID=Xetro.N01056.1.exon.4;Parent=Xetro.N01056.1;
scaffold_7	NO	CDS	54739128	54739193	.	+	0	ID=Xetro.N01056.1.CDS.4;Parent=Xetro.N01056.1;
scaffold_7	NO	exon	54743496	54743620	.	+	.	ID=Xetro.N01056.1.exon.5;Parent=Xetro.N01056.1;
scaffold_7	NO	CDS	54743496	54743576	.	+	0	ID=Xetro.N01056.1.CDS.5;Parent=Xetro.N01056.1;
scaffold_7	NO	three_prime_UTR	54743577	54743620	.	+	.	ID=Xetro.N01056.1.three_prime_UTR.1;Parent=Xetro.N01056.1;
scaffold_7	NO	gene	115809777	115815607	.	+	.	ID=Xetro.N02086;Name=rps19;
scaffold_7	NO	mRNA	115809777	115815607	.	+	.	ID=Xetro.N02086.1;Name=rps19.4;Parent=Xetro.N02086;
scaffold_7	NO	exon	115809777	115809792	.	+	.	ID=Xetro.N02086.1.exon.1;Parent=Xetro.N02086.1;
scaffold_7	NO	five_prime_UTR	115809777	115809792	.	+	.	ID=Xetro.N02086.1.five_prime_UTR.1;Parent=Xetro.N02086.1;
scaffold_7	NO	exon	115811890	115811960	.	+	.	ID=Xetro.N02086.1.exon.2;Parent=Xetro.N02086.1;
scaffold_7	NO	CDS	115811890	115811960	.	+	0	ID=Xetro.N02086.1.CDS.1;Parent=Xetro.N02086.1;
scaffold_7	NO	exon	115812266	115812366	.	+	.	ID=Xetro.N02086.1.exon.3;Parent=Xetro.N02086.1;
scaffold_7	NO	CDS	115812266	115812366	.	+	1	ID=Xetro.N02086.1.CDS.2;Parent=Xetro.N02086.1;
scaffold_7	NO	exon	115813536	115813719	.	+	.	ID=Xetro.N02086.1.exon.4;Parent=Xetro.N02086.1;
scaffold_7	NO	CDS	115813536	115813719	.	+	2	ID=Xetro.N02086.1.CDS.3;Parent=Xetro.N02086.1;
scaffold_7	NO	exon	115814235	115814289	.	+	.	ID=Xetro.N02086.1.exon.5;Parent=Xetro.N02086.1;
scaffold_7	NO	CDS	115814235	115814289	.	+	1	ID=Xetro.N02086.1.CDS.4;Parent=Xetro.N02086.1;
scaffold_7	NO	exon	115815541	115815607	.	+	.	ID=Xetro.N02086.1.exon.6;Parent=Xetro.N02086.1;
scaffold_7	NO	CDS	115815541	115815567	.	+	0	ID=Xetro.N02086.1.CDS.5;Parent=Xetro.N02086.1;
scaffold_7	NO	three_prime_UTR	115815568	115815607	.	+	.	ID=Xetro.N02086.1.three_prime_UTR.1;Parent=Xetro.N02086.1;
scaffold_8	NO	gene	70257390	70260820	.	+	.	ID=Xetro.N01430;Name=rps29;
scaffold_8	NO	mRNA	70257390	70260820	.	+	.	ID=Xetro.N01430.1;Name=rps29.1;Parent=Xetro.N01430;
scaffold_8	NO	exon	70257390	70257461	.	+	.	ID=Xetro.N01430.1.exon.1;Parent=Xetro.N01430.1;
scaffold_8	NO	exon	70258896	70258995	.	+	.	ID=Xetro.N01430.1.exon.2;Parent=Xetro.N01430.1;
scaffold_8	NO	exon	70260679	70260820	.	+	.	ID=Xetro.N01430.1.exon.3;Parent=Xetro.N01430.1;
scaffold_5	NO	gene	28896969	28907757	.	-	.	ID=Xetro.N00404;Name=rps7;
scaffold_5	NO	mRNA	28896969	28907757	.	-	.	ID=Xetro.N00404.1;Name=rps7.1;Parent=Xetro.N00404;
scaffold_5	NO	exon	28907724	28907757	.	-	.	ID=Xetro.N00404.1.exon.1;Parent=Xetro.N00404.1;
scaffold_5	NO	exon	28906304	28906385	.	-	.	ID=Xetro.N00404.1.exon.2;Parent=Xetro.N00404.1;
scaffold_5	NO	exon	28905193	28905264	.	-	.	ID=Xetro.N00404.1.exon.3;Parent=Xetro.N00404.1;
scaffold_5	NO	exon	28904489	28904632	.	-	.	ID=Xetro.N00404.1.exon.4;Parent=Xetro.N00404.1;
scaffold_5	NO	exon	28902425	28902489	.	-	.	ID=Xetro.N00404.1.exon.5;Parent=Xetro.N00404.1;
scaffold_5	NO	exon	28898100	28898250	.	-	.	ID=Xetro.N00404.1.exon.6;Parent=Xetro.N00404.1;
scaffold_5	NO	exon	28896969	28897100	.	-	.	ID=Xetro.N00404.1.exon.7;Parent=Xetro.N00404.1;
scaffold_6	NO	gene	99062223	99066780	.	-	.	ID=Xetro.N01284;Name=rpl15;
scaffold_6	NO	mRNA	99062223	99066780	.	-	.	ID=Xetro.N01284.1;Name=rpl15.3;Parent=Xetro.N01284;
scaffold_6	NO	exon	99066745	99066780	.	-	.	ID=Xetro.N01284.1.exon.1;Parent=Xetro.N01284.1;
scaffold_6	NO	five_prime_UTR	99066745	99066780	.	-	.	ID=Xetro.N01284.1.five_prime_UTR.1;Parent=Xetro.N01284.1;
scaffold_6	NO	exon	99065823	99066005	.	-	.	ID=Xetro.N01284.1.exon.2;Parent=Xetro.N01284.1;
scaffold_6	NO	CDS	99065823	99065994	.	-	0	ID=Xetro.N01284.1.CDS.1;Parent=Xetro.N01284.1;
scaffold_6	NO	five_prime_UTR	99065995	99066005	.	-	.	ID=Xetro.N01284.1.five_prime_UTR.2;Parent=Xetro.N01284.1;
scaffold_6	NO	exon	99064376	99064512	.	-	.	ID=Xetro.N01284.1.exon.3;Parent=Xetro.N01284.1;
scaffold_6	NO	CDS	99064376	99064512	.	-	2	ID=Xetro.N01284.1.CDS.2;Parent=Xetro.N01284.1;
scaffold_6	NO	exon	99062223	99062589	.	-	.	ID=Xetro.N01284.1.exon.4;Parent=Xetro.N01284.1;
scaffold_6	NO	three_prime_UTR	99062223	99062283	.	-	.	ID=Xetro.N01284.1.three_prime_UTR.1;Parent=Xetro.N01284.1;
scaffold_6	NO	CDS	99062284	99062589	.	-	0	ID=Xetro.N01284.1.CDS.3;Parent=Xetro.N01284.1;
scaffold_3	NO	gene	71973187	71980133	.	-	.	ID=Xetro.N01398;Name=rpl26;
scaffold_3	NO	mRNA	71973187	71980088	.	-	.	ID=Xetro.N01398.1;Name=rpl26.2;Parent=Xetro.N01398;
scaffold_3	NO	exon	71980064	71980088	.	-	.	ID=Xetro.N01398.1.exon.1;Parent=Xetro.N01398.1;
scaffold_3	NO	five_prime_UTR	71980064	71980088	.	-	.	ID=Xetro.N01398.1.five_prime_UTR.1;Parent=Xetro.N01398.1;
scaffold_3	NO	exon	71978389	71978561	.	-	.	ID=Xetro.N01398.1.exon.2;Parent=Xetro.N01398.1;
scaffold_3	NO	CDS	71978389	71978556	.	-	0	ID=Xetro.N01398.1.CDS.1;Parent=Xetro.N01398.1;
scaffold_3	NO	five_prime_UTR	71978557	71978561	.	-	.	ID=Xetro.N01398.1.five_prime_UTR.2;Parent=Xetro.N01398.1;
scaffold_3	NO	exon	71977452	71977592	.	-	.	ID=Xetro.N01398.1.exon.3;Parent=Xetro.N01398.1;
scaffold_3	NO	CDS	71977452	71977592	.	-	0	ID=Xetro.N01398.1.CDS.2;Parent=Xetro.N01398.1;
scaffold_3	NO	exon	71973187	71973348	.	-	.	ID=Xetro.N01398.1.exon.4;Parent=Xetro.N01398.1;
scaffold_3	NO	three_prime_UTR	71973187	71973219	.	-	.	ID=Xetro.N01398.1.three_prime_UTR.1;Parent=Xetro.N01398.1;
scaffold_3	NO	CDS	71973220	71973348	.	-	0	ID=Xetro.N01398.1.CDS.3;Parent=Xetro.N01398.1;
scaffold_5	NO	gene	8302934	8306784	.	+	.	ID=Xetro.N00125;Name=rpl35a;
scaffold_5	NO	mRNA	8302934	8306784	.	+	.	ID=Xetro.N00125.1;Name=rpl35a.1;Parent=Xetro.N00125;
scaffold_5	NO	exon	8302934	8302954	.	+	.	ID=Xetro.N00125.1.exon.1;Parent=Xetro.N00125.1;
scaffold_5	NO	five_prime_UTR	8302934	8302954	.	+	.	ID=Xetro.N00125.1.five_prime_UTR.1;Parent=Xetro.N00125.1;
scaffold_5	NO	exon	8304180	8304222	.	+	.	ID=Xetro.N00125.1.exon.2;Parent=Xetro.N00125.1;
scaffold_5	NO	five_prime_UTR	8304180	8304211	.	+	.	ID=Xetro.N00125.1.five_prime_UTR.2;Parent=Xetro.N00125.1;
scaffold_5	NO	CDS	8304212	8304222	.	+	0	ID=Xetro.N00125.1.CDS.1;Parent=Xetro.N00125.1;
scaffold_5	NO	exon	8304967	8305119	.	+	.	ID=Xetro.N00125.1.exon.3;Parent=Xetro.N00125.1;
scaffold_5	NO	CDS	8304967	8305119	.	+	1	ID=Xetro.N00125.1.CDS.2;Parent=Xetro.N00125.1;
scaffold_5	NO	exon	8306012	8306784	.	+	.	ID=Xetro.N00125.1.exon.4;Parent=Xetro.N00125.1;
scaffold_5	NO	CDS	8306012	8306213	.	+	1	ID=Xetro.N00125.1.CDS.3;Parent=Xetro.N00125.1;
scaffold_5	NO	three_prime_UTR	8306214	8306784	.	+	.	ID=Xetro.N00125.1.three_prime_UTR.1;Parent=Xetro.N00125.1;
scaffold_5	NO	mRNA	8302934	8306784	.	+	.	ID=Xetro.N00125.2;Name=rpl35a.2;Parent=Xetro.N00125;
scaffold_5	NO	exon	8302934	8304222	.	+	.	ID=Xetro.N00125.2.exon.1;Parent=Xetro.N00125.2;
scaffold_5	NO	five_prime_UTR	8302934	8304211	.	+	.	ID=Xetro.N00125.2.five_prime_UTR.1;Parent=Xetro.N00125.2;
scaffold_5	NO	CDS	8304212	8304222	.	+	0	ID=Xetro.N00125.2.CDS.1;Parent=Xetro.N00125.2;
scaffold_5	NO	exon	8304967	8305119	.	+	.	ID=Xetro.N00125.2.exon.2;Parent=Xetro.N00125.2;
scaffold_5	NO	CDS	8304967	8305119	.	+	1	ID=Xetro.N00125.2.CDS.2;Parent=Xetro.N00125.2;
scaffold_5	NO	exon	8306012	8306156	.	+	.	ID=Xetro.N00125.2.exon.3;Parent=Xetro.N00125.2;
scaffold_5	NO	CDS	8306012	8306156	.	+	1	ID=Xetro.N00125.2.CDS.3;Parent=Xetro.N00125.2;
scaffold_5	NO	exon	8306716	8306784	.	+	.	ID=Xetro.N00125.2.exon.4;Parent=Xetro.N00125.2;
scaffold_5	NO	CDS	8306716	8306739	.	+	0	ID=Xetro.N00125.2.CDS.4;Parent=Xetro.N00125.2;
scaffold_5	NO	three_prime_UTR	8306740	8306784	.	+	.	ID=Xetro.N00125.2.three_prime_UTR.1;Parent=Xetro.N00125.2;
scaffold_5	NO	mRNA	8302934	8306784	.	+	.	ID=Xetro.N00125.3;Name=rpl35a.3;Parent=Xetro.N00125;
scaffold_5	NO	exon	8302934	8303420	.	+	.	ID=Xetro.N00125.3.exon.1;Parent=Xetro.N00125.3;
scaffold_5	NO	five_prime_UTR	8302934	8303420	.	+	.	ID=Xetro.N00125.3.five_prime_UTR.1;Parent=Xetro.N00125.3;
scaffold_5	NO	exon	8304180	8304222	.	+	.	ID=Xetro.N00125.3.exon.2;Parent=Xetro.N00125.3;
scaffold_5	NO	five_prime_UTR	8304180	8304211	.	+	.	ID=Xetro.N00125.3.five_prime_UTR.2;Parent=Xetro.N00125.3;
scaffold_5	NO	CDS	8304212	8304222	.	+	0	ID=Xetro.N00125.3.CDS.1;Parent=Xetro.N00125.3;
scaffold_5	NO	exon	8304967	8305119	.	+	.	ID=Xetro.N00125.3.exon.3;Parent=Xetro.N00125.3;
scaffold_5	NO	CDS	8304967	8305119	.	+	1	ID=Xetro.N00125.3.CDS.2;Parent=Xetro.N00125.3;
scaffold_5	NO	exon	8306012	8306156	.	+	.	ID=Xetro.N00125.3.exon.4;Parent=Xetro.N00125.3;
scaffold_5	NO	CDS	8306012	8306156	.	+	1	ID=Xetro.N00125.3.CDS.3;Parent=Xetro.N00125.3;
scaffold_5	NO	exon	8306716	8306784	.	+	.	ID=Xetro.N00125.3.exon.5;Parent=Xetro.N00125.3;
scaffold_5	NO	CDS	8306716	8306739	.	+	0	ID=Xetro.N00125.3.CDS.4;Parent=Xetro.N00125.3;
scaffold_5	NO	three_prime_UTR	8306740	8306784	.	+	.	ID=Xetro.N00125.3.three_prime_UTR.1;Parent=Xetro.N00125.3;
scaffold_5	NO	mRNA	8302934	8306784	.	+	.	ID=Xetro.N00125.4;Name=rpl35a.4;Parent=Xetro.N00125;
scaffold_5	NO	exon	8302934	8303443	.	+	.	ID=Xetro.N00125.4.exon.1;Parent=Xetro.N00125.4;
scaffold_5	NO	five_prime_UTR	8302934	8303443	.	+	.	ID=Xetro.N00125.4.five_prime_UTR.1;Parent=Xetro.N00125.4;
scaffold_5	NO	exon	8304180	8304222	.	+	.	ID=Xetro.N00125.4.exon.2;Parent=Xetro.N00125.4;
scaffold_5	NO	five_prime_UTR	8304180	8304211	.	+	.	ID=Xetro.N00125.4.five_prime_UTR.2;Parent=Xetro.N00125.4;
scaffold_5	NO	CDS	8304212	8304222	.	+	0	ID=Xetro.N00125.4.CDS.1;Parent=Xetro.N00125.4;
scaffold_5	NO	exon	8304967	8305119	.	+	.	ID=Xetro.N00125.4.exon.3;Parent=Xetro.N00125.4;
scaffold_5	NO	CDS	8304967	8305119	.	+	1	ID=Xetro.N00125.4.CDS.2;Parent=Xetro.N00125.4;
scaffold_5	NO	exon	8306012	8306156	.	+	.	ID=Xetro.N00125.4.exon.4;Parent=Xetro.N00125.4;
scaffold_5	NO	CDS	8306012	8306156	.	+	1	ID=Xetro.N00125.4.CDS.3;Parent=Xetro.N00125.4;
scaffold_5	NO	exon	8306716	8306784	.	+	.	ID=Xetro.N00125.4.exon.5;Parent=Xetro.N00125.4;
scaffold_5	NO	CDS	8306716	8306739	.	+	0	ID=Xetro.N00125.4.CDS.4;Parent=Xetro.N00125.4;
scaffold_5	NO	three_prime_UTR	8306740	8306784	.	+	.	ID=Xetro.N00125.4.three_prime_UTR.1;Parent=Xetro.N00125.4;
scaffold_5	NO	mRNA	8302934	8306784	.	+	.	ID=Xetro.N00125.5;Name=rpl35a.5;Parent=Xetro.N00125;
scaffold_5	NO	exon	8302934	8302954	.	+	.	ID=Xetro.N00125.5.exon.1;Parent=Xetro.N00125.5;
scaffold_5	NO	five_prime_UTR	8302934	8302954	.	+	.	ID=Xetro.N00125.5.five_prime_UTR.1;Parent=Xetro.N00125.5;
scaffold_5	NO	exon	8303053	8303420	.	+	.	ID=Xetro.N00125.5.exon.2;Parent=Xetro.N00125.5;
scaffold_5	NO	five_prime_UTR	8303053	8303420	.	+	.	ID=Xetro.N00125.5.five_prime_UTR.2;Parent=Xetro.N00125.5;
scaffold_5	NO	exon	8304180	8304222	.	+	.	ID=Xetro.N00125.5.exon.3;Parent=Xetro.N00125.5;
scaffold_5	NO	five_prime_UTR	8304180	8304211	.	+	.	ID=Xetro.N00125.5.five_prime_UTR.3;Parent=Xetro.N00125.5;
scaffold_5	NO	CDS	8304212	8304222	.	+	0	ID=Xetro.N00125.5.CDS.1;Parent=Xetro.N00125.5;
scaffold_5	NO	exon	8304967	8305119	.	+	.	ID=Xetro.N00125.5.exon.4;Parent=Xetro.N00125.5;
scaffold_5	NO	CDS	8304967	8305119	.	+	1	ID=Xetro.N00125.5.CDS.2;Parent=Xetro.N00125.5;
scaffold_5	NO	exon	8306012	8306156	.	+	.	ID=Xetro.N00125.5.exon.5;Parent=Xetro.N00125.5;
scaffold_5	NO	CDS	8306012	8306156	.	+	1	ID=Xetro.N00125.5.CDS.3;Parent=Xetro.N00125.5;
scaffold_5	NO	exon	8306716	8306784	.	+	.	ID=Xetro.N00125.5.exon.6;Parent=Xetro.N00125.5;
scaffold_5	NO	CDS	8306716	8306739	.	+	0	ID=Xetro.N00125.5.CDS.4;Parent=Xetro.N00125.5;
scaffold_5	NO	three_prime_UTR	8306740	8306784	.	+	.	ID=Xetro.N00125.5.three_prime_UTR.1;Parent=Xetro.N00125.5;
scaffold_5	NO	mRNA	8302934	8306784	.	+	.	ID=Xetro.N00125.6;Name=rpl35a.6;Parent=Xetro.N00125;
scaffold_5	NO	exon	8302934	8302954	.	+	.	ID=Xetro.N00125.6.exon.1;Parent=Xetro.N00125.6;
scaffold_5	NO	five_prime_UTR	8302934	8302954	.	+	.	ID=Xetro.N00125.6.five_prime_UTR.1;Parent=Xetro.N00125.6;
scaffold_5	NO	exon	8304180	8304222	.	+	.	ID=Xetro.N00125.6.exon.2;Parent=Xetro.N00125.6;

scaffold_5	NO	five_prime_UTR	8304180	8304211	.	+	.	ID=Xetro.N00125.6.five_prime_UTR.2;Parent=Xetro.N00125.6;
scaffold_5	NO	CDS	8304212	8304222	.	+	0	ID=Xetro.N00125.6.CDS.1;Parent=Xetro.N00125.6;
scaffold_5	NO	exon	8304967	8305119	.	+	.	ID=Xetro.N00125.6.exon.3;Parent=Xetro.N00125.6;
scaffold_5	NO	CDS	8304967	8305119	.	+	1	ID=Xetro.N00125.6.CDS.2;Parent=Xetro.N00125.6;
scaffold_5	NO	exon	8306012	8306156	.	+	.	ID=Xetro.N00125.6.exon.4;Parent=Xetro.N00125.6;
scaffold_5	NO	CDS	8306012	8306156	.	+	1	ID=Xetro.N00125.6.CDS.3;Parent=Xetro.N00125.6;
scaffold_5	NO	exon	8306716	8306784	.	+	.	ID=Xetro.N00125.6.exon.5;Parent=Xetro.N00125.6;
scaffold_5	NO	CDS	8306716	8306739	.	+	0	ID=Xetro.N00125.6.CDS.4;Parent=Xetro.N00125.6;
scaffold_5	NO	three_prime_UTR	8306740	8306784	.	+	.	ID=Xetro.N00125.6.three_prime_UTR.1;Parent=Xetro.N00125.6;
scaffold_29	NO	gene	22976	27272	.	-	.	ID=Xetro.N00388;Name=rpl38;
scaffold_29	NO	mRNA	22976	27272	.	-	.	ID=Xetro.N00388.1;Name=rpl38.1;Parent=Xetro.N00388;
scaffold_29	NO	exon	27243	27272	.	-	.	ID=Xetro.N00388.1.exon.1;Parent=Xetro.N00388.1;
scaffold_29	NO	exon	27056	27085	.	-	.	ID=Xetro.N00388.1.exon.2;Parent=Xetro.N00388.1;
scaffold_29	NO	exon	25680	25740	.	-	.	ID=Xetro.N00388.1.exon.3;Parent=Xetro.N00388.1;
scaffold_29	NO	exon	24571	24693	.	-	.	ID=Xetro.N00388.1.exon.4;Parent=Xetro.N00388.1;
scaffold_29	NO	exon	22976	23041	.	-	.	ID=Xetro.N00388.1.exon.5;Parent=Xetro.N00388.1;
