# Supplementary figures and images for: Expression of ribosomopathy genes during Xenopus tropicalis embryogenesis
Source: BMC Dev Biol. 2016 Oct 26;16:38. doi: 10.1186/s12861-016-0138-5 (PMC5081970; doi:10.1186/s12861-016-0138-5)

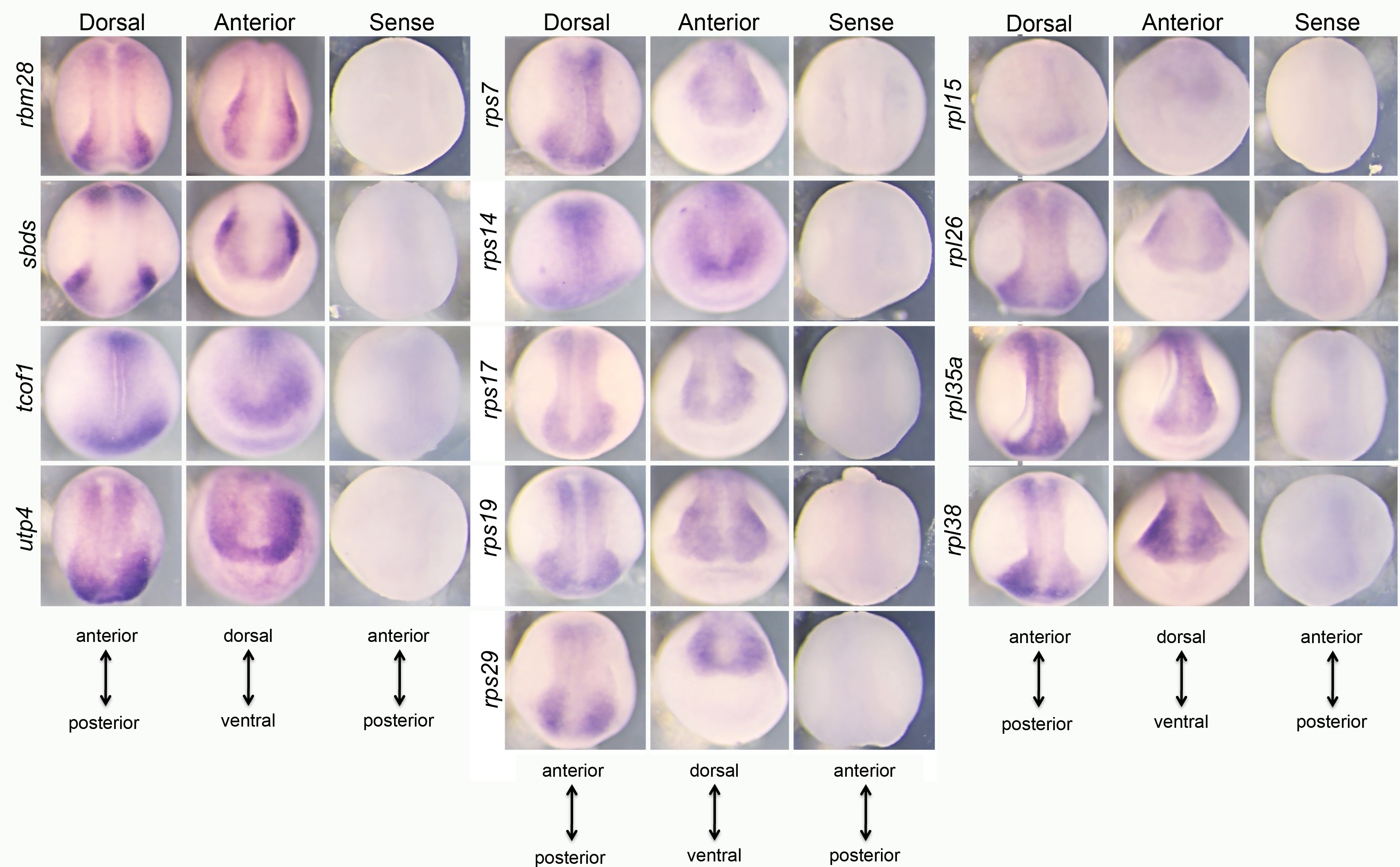

Supplement: Additional file 2: — Comparison of digoxigenin labeled anti-sense and sense mRNA probes for each ribosomopathy gene in stage 16 embryos. Dorsal and anterior views are shown. Note the strong expression detected in the neural plate by the anti-sense mRNA probes while the control sense probed embryos remains unstained. (JPG 922 kb) [file 12861_2016_138_MOESM2_ESM.jpg]
